# Secondary structure assignment that accurately reflects physical and evolutionary characteristics

**DOI:** 10.1186/1471-2105-6-S4-S8

**Published:** 2005-12-01

**Authors:** Maria Vittoria Cubellis, Fabien Cailliez, Simon C Lovell

**Affiliations:** 1 Biochemistry Dept, University of Cambridge, Cambridge CB2 1GA, UK; 2 Dipartimento di Biologia Strutturale e Funzionale, Napoli, Italy; 3 Institut de Biologie Physico-Chimique, Paris, France; 4 Faculty of Life Sciences, University of Manchester, Manchester, UK

## Abstract

**Background:**

Secondary structure is used in hierarchical classification of protein structures, identification of protein features, such as helix caps and loops, for fold recognition, and as a precursor to ab initio structure prediction. There are several methods available for assigning secondary structure if the three-dimensional structure of the protein is known. Unfortunately they differ in their definitions, particularly in the exact positions of the termini. Additionally, most existing methods rely on hydrogen bonding, which means that important secondary structural classes, such as isolated β-strands and poly-proline helices cannot be identified as they do not have characteristic hydrogen-bonding patterns. For this reason we have developed a more accurate method for assigning secondary structure based on main chain geometry, which also allows a more comprehensive assignment of secondary structure.

**Results:**

We define secondary structure based on a number of geometric parameters. Helices are defined based on whether they fit inside an imaginary cylinder: residues must be within the correct radius of a central axis. Different types of helices (alpha, 3_10 _or π) are assigned on the basis of the angle between successive peptide bonds. β-strands are assigned based on backbone dihedrals and with alternating peptide bonds. Thus hydrogen bonding is not required and β-strands can be within a parallel sheet, antiparallel sheet, or can be isolated. Poly-proline helices are defined similarly, although with three-fold symmetry.

**Conclusion:**

We find that our method better assigns secondary structure than existing methods. Specifically, we find that comparing our methods with those of others, amino-acid trends at helix caps are stronger, secondary structural elements less likely to be concatenated together and secondary structure guided sequence alignment is improved. We conclude, therefore, that secondary structure assignments using our method better reflects physical and evolutionary characteristics of proteins.

The program is available from

## Background

Secondary structure in proteins is an important level in the hierarchical classification of structure. It is not only a convenient tool to simplify the description of protein structure, but it also reflects physical principles of folding. Secondary structure is used in the classification of structure [1-3], the classification of protein features [4-6], in the assignment of local environments for fold/homology recognition techniques [7,8], and for the study of secondary structure itself [9,10]. The prediction of secondary structure, both for its own ends [11] and as a precursor to *ab inito *protein structure prediction, is an active field [12]. All of these techniques rely on the accurate assignment of secondary structure. For some applications, such as fold classification, the details of secondary structure assignment are less important than the general features, but for some applications, particularly the study of features near the end of structures, the exact assignment of the secondary structure termini are vital.

Accurate assignment of secondary structure may be viewed as somewhat arbitrary. After all, different experts may disagree on the details of secondary structure assignment. However, the polypeptide chain has different physical characteristics in different secondary structure types – for example different sequence preferences. An optimal assignment of secondary structure will be one that matches not only our understanding of various secondary structure types, but also reflects these physical characteristics.

Secondary structures may be characterized by a certain geometry which is the consequence of a network of hydrogen bonds between the > C = O group of residue n and the >N-H group of another residue m. For example in an α-helix m = n+4 and in a 3_10 _helix m = n+3. The presence of hydrogen bonds has often been exploited to develop algorithms assigning secondary structure elements based on the calculation of hydrogen bond energies [13,14]. Some other programs use geometric recognition of secondary structures [15-18]. The geometric features employed are numerous and quite different. The program xtlsstruc [15] for example uses the angles between three consecutive Cα atoms, the dihedral angle between two consecutive carbonyl groups and distances between atoms to determine helices and strands. The program P-curve [17] is based on an algorithm defining an axis along the protein and determines the structures using parameters relative to this axis. However the programs which are the most widely used are Stride and especially DSSP based both on the calculation of hydrogen bonds.

DSSP [14] calculates energies of hydrogen bonds using a classical electrostatic function. The residues are assigned in a secondary structure category depending on their main chain hydrogen bonding.

Stride [13] also calculates energies of hydrogen bonds but uses a different function which also takes into account backbone torsion angles. This results in the elimination of many of the false positives, although no restriction is placed on φ/ψ angles for 3_10 _helices. Although Stride can be considered as an improvement on DSSP, it can also produce incorrect assignments in some cases.

The obligation to be involved in two hydrogen bonds for a residue in the middle of a secondary structure is very restrictive. Distortions frequently cause individual hydrogen bonds to be missing, or made in a non-canonical manner. Used in an unmodified form to assign secondary structure the hydrogen bonding energy function results in a large number of artificially short secondary structures. To reduce this problem it is necessary to gather together two secondary structural elements that have an overlap according to the definition of elements by the algorithm [14]. Unfortunately, this approach can overcompensate, and produce artificially long helices and strands, either by merging two separate secondary structural elements into a single long secondary structure, or incorrectly extending a secondary structure past its true terminus. For these reasons we chose a method which, in the main, does not use hydrogen bonding considerations. An additional advantage of using a definition that does not depend on hydrogen bonding is that it allows the assignment of secondary structures that do not have characteristic hydrogen bonding patterns, such as isolated β-strands and poly-proline helices.

We have decided to develop a new algorithm based on geometric features to assign secondary structures, incorporated in a new program called SEGNO. We think that this geometric approach can produce improvements on the previous methods. Specifically, we show that our new definition leads to better correlations with physical and evolutionary characteristics of proteins.

## Results & discussion

Segno assigns each residue of a protein into one of the following categories: α-helix, 3_10 _helix, π-helix, poly-proline helix or β-strand. Residues that fall into none of these categories are given the assignment "coil". These residues are then grouped together to form secondary structural elements of the following categories: α-helices, 3_10 _helices, π-helices, mixed helices (containing a combination of α, 3_10 _and/or π residues), isolated β-strands, β-strands belonging to a β-sheet and poly-proline helices. 3_10 _helices, b-strands and poly-proline helices must contain at least three residues, π-helices at least four residues, and π-helices at least five.

Benchmarking secondary structure assignment programs is not straightforward. It used to be possible to benchmark against hand assignments made by x-ray crystallographers and NMR spectroscopists as given in PDB files. An accurate assignment was one that agreed with human assignments [13]. More recently there has been routine automatic use of a DSSP-like algorithm by the PDB, which makes this impossible. Any differences between SEGNO and the PDB assignments would merely be differences between the two algorithms, with no objective "correct" answer. We have chosen, therefore, to use our algorithm in a number of applications that are sensitive to accurate secondary structure assignment. We conclude that our program is more or less accurate than current techniques if it is more or less useful in a wide range of situations. This approach is more objective than it may first appear. If a secondary structure assignment makes clearer particular features of protein structure it may be argued that this assignment more accurately reflects the physical or evolutionary restraints imposed on the protein structure.

The degree of agreement between the three programs tested is as follows: SEGNO and STRIDE, 84.1%; SEGNO and DSSP, 82.4%; DSSP and STRIDE, 81.0%.

### Helix Capping

Helix caps were originally defined as the first or last residue within an α-helix (for N- and C-caps, respectively) [5,6]. There are several N-capping motifs, reviewed by Aurora and Rose [19]. The most common motif consists of a hydrogen bond between the oxygen of the side chain of the N-cap residue (n) and the >N-H group of the residue n+3 (the third residue of the helix). The local structural environment requires particular amino-acids and specific structural features at and near the helix caps, and so methods of assigning secondary structure can be evaluated using these residue preferences. Specifically, a method of assigning secondary structure may be classed as superior if it gives stronger position-specific amino-acid preferences [5].

The residues which can adopt the correct geometry to form the N-cap hydrogen bond are serine, threonine, asparagine or aspartate. For N-caps we determined the occurrence of these residues at the helix N-terminii as defined by SEGNO, DSSP and STRIDE. Residues were only counted as N-caps if they made the required hydrogen bond from the side chain of residue i to the main chain NH of residue i+3.

At the C-termini of helices specific sequence and structural motifs often occur [10]. Helix C-caps often have a residue with positive φ, which allows the chain to turn back and satisfy hydrogen bond acceptors, known as the "Schellman motif" [20]. We therefore identify residues with positive φ near the C-terminal end of the helix, as defined by all three programs.

Residue preferences for N-caps and structural preferences for C-caps for secondary structure elements in the database of 500 structures are given in tables 1 and 2.

The distribution of the position of the first residue with a positive φ shows in the three cases a peak for the position 0. However that peak is sharper for SEGNO and STRIDE, suggesting a more reliable definition of C-terminal ends of helices. We can see that we have a much greater number of helices where the C-cap residue is counted as the last residue of the helix for SEGNO and STRIDE, while with DSSP the position of the C-cap residue is more widely spread over the different positions at the end of the helix. Moreover we can see that with DSSP a proportion of residues with a positive φ are found in helices which is incompatible with the backbone dihedral angles of a residue in an a helix. In contrast there are no residues with positive φ at the C-terminus end of helices with SEGNO or STRIDE α-helical assignments.

### Secondary Structure Distortions

Idealized helices and strands are straight, but in the reality secondary structures that occur in proteins have a number of distortions, including bends. These bends may be due to many factors (steric interaction between side chains, interaction with solvent molecules [21]). However bends are very rarely large in size. In contrast, mis-assignment of secondary structures can result in apparently large bends, for example in helices where a helix-turn-helix or strand-turn-strand combination is assigned as a single element. Thus a large number of extreme bends should be viewed with suspicion.

**Figure 1:**
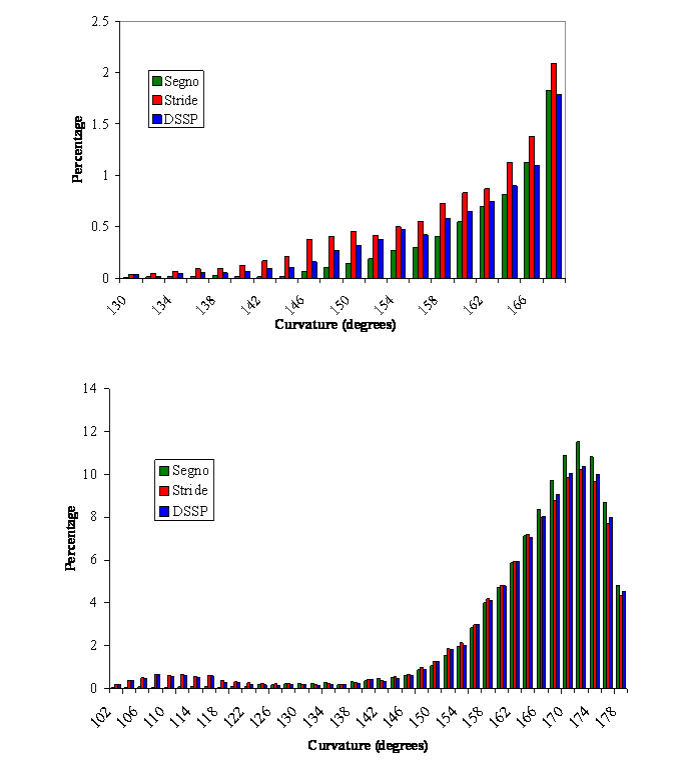
Curvature in secondary structures for (a) α-helices and (b) β-strands for the three programs' definitions. The x-axis represents curvature in degrees in a given bin, with the y-axis representing the percentage of residues in that bin. In (a) the bins >170° are omitted to show the distribution in the more distorted region more clearly.

**Figure 2:**
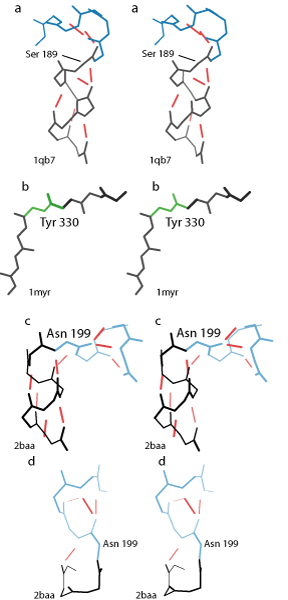
Examples of secondary structural elements showing extreme curvature, which are most likely to be due to mis-assignment of the secondary structure (a) 1qb7 (b) 1myr (c) and (d) 2baa. (c) and (d) show different views of the same region of the protein. In all cases all residues shown are assigned to be in continuous secondary structures by DSSP and STRIDE. SEGNO assignments are shown in black. Hydrogen bonds are shown in red. Figures are shown as stereo pairs.

For both helices and β-strands we superimposed an ideal secondary structural element. The bend at residue n is defined as the angle between the axis of the ideal element of superimposed on the residue n-1 and the axis of the ideal element superimposed on the residue n+1, with 180° representing a straight element. It can be seen from figure 1 that SEGNO assigns helices and strands with less extreme bends than STRIDE and DSSP. For helices (figure 1a) this is particularly remarkable in the region of bends between 125 and 165°, which correspond to very bent helices. For strands there is a marked peak in the DSSP and STRIDE distributions around 110°, which is not seen in the SEGNO distribution. We have examined all examples with extreme distortions (angles more acute than 160° for helices and 140° for strands). We find that in all cases we disagree with the secondary structure assignment: all extreme distortions we observe arise from secondary structure assignments extending beyond the true termini of the helix or strand. Examples are shown in figure 2.

In extreme cases it is possible for the chain to bend back on itself at the end of a helix to form a loop with several i to i+4 hydrogen bonds. Both DSSP and STRIDE mis-assign these residues as helical (figure 2c and 2d) even though they are clearly not. In the example shown a single residue (asn 199) has non-helical φ/ψ angles which makes the chain turn and ends the helix. Two residues are missing hydrogen bonds. SEGNO appropriately assigns the helix end in this case.

### Secondary structure guided sequence alignment

Because protein structure is more conserved than sequence, secondary structure can be used to improve sequence alignment quality when the structure of one of the proteins is known. This approach has been widely used for fold recognition [7,8]. If the secondary structure assignment is incorrect, the alignment guided by this assignment will be degraded.

**Figure 3:**
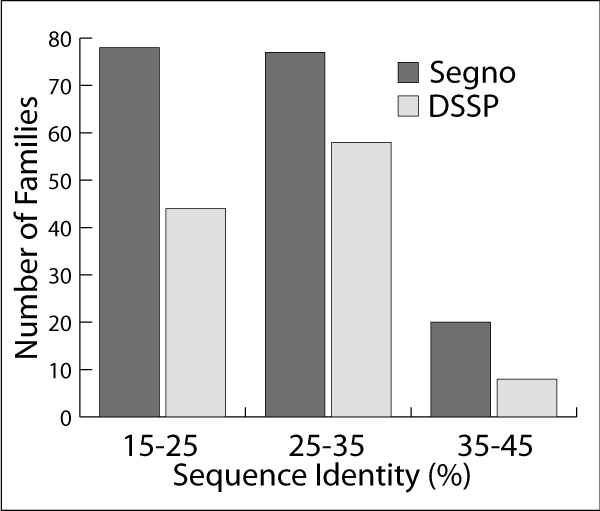
Secondary-structure guided sequence alignments for SEGNO and DSSP over a range of sequence similarities. Bars indicate proportion of cases where SEGNO gives a more accurate alignment than DSSP (dark bars) or where DSSP gives a more accurate alignment than SEGNO (light bars). Total number of observations in each bin are: 15–25%, 122; 25–35%, 135; 35–45%, 28.

In order to test whether SEGNO secondary structure assignments improve structure-guided sequence alignment, we took families from the HOMSTRAD database [3]. We assigned secondary structure to one of the protein structures using DSSP or SEGNO, and used FUGUE [7] to align the sequence of the other family member. We then superimposed the two protein structures, using this sequence alignment as the set of equivalent residues. If the sequence alignment is correct structurally equivalent residues should be aligned, and so the root mean square deviation (RMSD) will be low. Conversely errors in the alignment will result in non-equivalent secondary structures and higher RMSD. Results for a set of two-member families from HOMSTRAD are shown in figure 3. Overall DSSP gives better alignments than SEGNO for 110 families (39%), whereas SEGNO shows improvements over DSSP for 175 families (61%). The improvement in alignment quality does not correlate with sequence divergence, which is perhaps surprising, given that the contribution from secondary to alignment quality is more important for more divergent sequences.

## Conclusion

To a certain extent, secondary structure may be viewed as a human construction. Our assignment of it depends on our own definition of it, leading to a somewhat circular argument. Previous authors have validated their method by showing that it corresponds more exactly to human expert assignment [13]. This is no longer possible, as the PDB now automatically assigns secondary structure using DSSP. However, it should be realised that secondary structure exists in proteins due to the physical characteristics of the polypeptide chain. Specifically it arises because the polar backbone must pass though the low-dielectric protein core. Consequently, it would be highly energetically unfavourable to leave the backbone hydrogen bond donors and acceptors unsatisfied. Due to the geometry of the peptide backbone there are only two repeating ways of satisfying hydrogen bonds without giving rise to van der Waals overlaps: the α-helix and the β-sheet.

The polypeptide chain in the various types of secondary structure has different physical characteristics. For example, β-branched amino acids are over-represented in β-strands [22], residues near the ends of helices have clear residue preferences to make helix caps [5,6]. An assignment algorithm that reflects these physical properties is one that more accurately describes the structure. Similarly, if the secondary structure is mis-assigned when producing structure-based alignments, incorrect alignments can arise. Furthermore, if these alignments are used to generate environment-specific substitution tables [23,24], clearly the environments must be correctly assigned.

In the majority of the cases the assignments provided by the various assignment programs are similar (more than 80%). However a further analysis of the results has revealed that this apparent agreement hides many differences particularly in the definition of the end of the structures.

When examining the sequence preferences at the ends of helices, SEGNO and STRIDE perform approximately equivalently, and give rise to sequence preferences that are clearer than DSSP. When the assignments of distorted secondary structures are inspected, SEGNO makes assignments that stop at the boundaries of secondary structure, and are therefore more reliable. STRIDE and DSSP have a tendency to read through non-secondary structure regions, producing artificially-distorted secondary structures. If the study of distortions in secondary structures is the aim, then accurate assignment, with minimal running-together of truly separate secondary structures is essential.

An additional advantage of using a geometric description of secondary structure is that it allows the assignment of isolated β-strands and poly-proline helices (4% and 3% of residues, respectively). These structures do not make regular patterns of hydrogen bonds and cannot, therefore, be identified by hydrogen bonding functions. These structures are somewhat unusual in that they have their main chain hydrogen bond donors and acceptors unsatisfied at least by other local regular main chain interactions. We have found that poly-proline helices are often found in protein-protein interaction sites [25], probably because their unsatisfied hydrogen bond donors and acceptors can be "read" by interacting proteins [26]. The same is true for isolated β-strands. Thus our new method allows investigation of these biologically important protein elements.

## Materials and methods

The program SEGNO uses geometric parameters to define secondary structure. We were inspired by a paper published by Richardson and Richardson [5] in which they characterised residue preferences at the termini of α-helices. They used a geometric description of helices, in which the first residue that leaves an imaginary cylinder projected along the helix is defined as the capping residue. Since a cylinder can be defined by an axis and a radius, this is the approach used to defined helices. This technique was adapted for other secondary structural elements.

The axis of the structures is approximated by calculating the mean three-dimensional coordinate of a window of four Cα positions. Although this gives only an approximate axis, it has the advantage that it does not require prior knowledge of the secondary structure in contrast to other methods for determining the local axis of secondary structure, for example [9]. Because the approximate axis is defined only on four Cα atoms, it can distort as the local secondary structure does, making the assignment robust towards secondary structure distortions. Secondary structure was assigned by distance from the axis to the appropriate Cα coordinate, and the angle τ described by the local axis and this vector. The dihedral angle between the peptide plain of residue i and residue i+n was used as a constraint, as discussed below. This parameter was termed ωn and was calculated for values of n from 2 to 5, as appropriate. Additionally the backbone dihedral angle φ and ψ are also used. The use of backbone dihedral angles ensures both the accuracy of the definition of the ends of secondary structure and the correct handedness, filtering out, for example, left-handed helices.

Cut-offs for all parameters used for assigning secondary structure were determined empirically. To do this, authors assigned secondary structure by visual inspection, and adjusted parameters until the automatic assignments matched the manual ones. Dihedral angle parameters, for example, φ and ψ, were defined to a precision of no more than 5°. Once cut-offs were determined the subsequent tests for accuracy were performed (see results section) with no further adjustment of parameters.

## Recognition of helical residues

Initially, residues are recognised as helical, and subsequently assigned to either α, 3_10 _or π classes. Poly-proline helices are more similar to β-strands, and will be discussed below.

In order to be defined as a helix, (1) the radius (denoted r) must be between 1.7 and 3.0 Å, (2) τ must be between 75 and 120°, (3) φ must be between -95 and -35°, (4) ψ must be between -70 and -10°.

Although these cut-offs are not overly strict we have additional problems at the C-termini of the helices. These problems have two different origins. Firstly, at the end of a helix the axis defined by the mean position of Cα carbons is not as close to the real axis as it is in the middle of the helix because it contains information from non-helical residues. Thus the angle made by the radius and the axis for the three last helical residues may not be in the range of the cut-offs. The algorithm therefore calculates the complementary angle of τ (termed τ-1), which must define a set of complementary cut-offs. The second problem is that the C-termini ends of helices are more variable than the other helical residues. The reason for this is that the four last helical residues often participate at only one hydrogen bond, whereas the other helical residues participate in two, including the first residues that are very often engaged in a hydrogen bond with side chains. We therefore used less constrained cut-offs for the last three residues of the helix in order to assign them correctly (50 ≤ τ-1 ≤ 112 which corresponds to 68 ≤ τ ≤ 130).

### Distinguishing different types of helices

Once a residue has been assigned as a helix, its type (α, 3_10 _or π) is determined. As certain parameters cannot be calculated for short helices the details differ with helix length.

A 3_10 _helix completes a complete turn in 3 residues, an α-helix in 4 and a π-helix in 5 residues. Accordingly, if the distances between the carbonyl oxygen atoms and peptide nitrogen atoms along the chain, for 3_10 _helices the O-N distance between residues i and i+3 will be shorter than the O-N distance between residues i and i+4. For α-helices the reverse is true. For π-helices the i to i+5 distance will be shorter than i to i+4 or i to i+3. It should be noted that determining this distance is equivalent to calculating a hydrogen bond, and so an absolute cut off of 3.5 Å was also applied.

For helices of 3 or more residues, the dihedral angle between the peptide bond of residue i and residue i+3 (termed ω3), residue i and i+4 (ω4) and i to i+5 (ω5) was calculated. In the case of a 3_10 _residue ω3 is closer to 180° and thus is assigned as 3_10 _if ω3 > ω4. A residue is assigned as alpha if ω3<ω4 and ω4>ω5. A residue is assigned as π helix if ω4<ω5. In each case the φ and ψ angles must also be appropriate for the assigned structure.

### Recognition of beta strands and sheets

We use the term β-strand to mean a single contiguous piece of the polypeptide chain in β-conformation. A β-sheet is made by several β-strands connected by hydrogen bonds.

The recognition of β-strands is based on four parameters: the angle τ, the dihedral angle between the amide plains of i and i+1 (called ω1), φ, and ψ. To determine if the residue i is in a β-strand with the residue i+1, τ must be greater than 110°, ω 1 must be between 123 and 210°, and φ(i+1) and ψ(i) must be inside the region of a beta strand in the Ramachandran plot (170° < φ < 290°, 60°<ψ<185°; in all cases dihedral angles ranges are given in the most convenient reference frame to represent where the value lies. Values >180° can be converted into the usual range of -180° to +180° by the addition of 360°). To determine if the residue i is in a strand with the residue i-1, ω-1 must be less than 80°, ω-1(the dihedral angle between the carbonyl group n and the carbonyl group n-1) must be between 125 and 210°, and φ(i) and ψ(i) must be inside the region of a β-strand in the Ramachandran plot (as defined above). Finally the strands with less than three residues are eliminated.

Strands are associated into sheets if they have at least two inter-strand hydrogen bonds (O to N distance of <4Å), and are approximately parallel (the dihedral between the strand axes >135°).

### Definition of Poly-proline helices

Poly-proline helices were defined as previously published [25]. Briefly, we measured 4 dihedral angles: φ,ψ, diheco (the dihedral angle between O(i-1), C(i-1), C(i), O(i) where i represents the residue number) and diheco2 (the dihedral angle between O(i-1), C(i-1), C(i+1), O(i+1)). The two dihedral angles diheco and diheco2 represent the angles between the planes of successive peptide bonds, separated by one and two residues respectively.

We temporarily assign a residue to a poly-proline conformation if it has not been previously assigned to b-strand by SEGNO and if: φ is -125° to -35°; ψ is 100 to 185; diheco is 180° to 300° and diheco2 is 80° to 160°. That is, if φ and ψ have appropriate values, and if there is approximate three-fold symmetry in the poly-proline helix. We maintain the assignment only for those stretches of residues that have an average diheco in the range 220°–270 and an average diheco2 in the range 100°–140°. Less strict φ and ψ restraints are used for the residues at the end of poly-proline stretches: φ for the last residue in a PPII helix is allowed to be in the range 90–195°, whereas ψ for the first residue of poly-proline helix is allowed to be in the range -145° to -60°. Deviations of the first and the last dihedral angles in the stretch, in fact, do not influence the left-handed helical structure with the overall shape resembling a triangular prism. Poly-proline helices have a minimal length of 3.

### Length Constraint and Ramachandran constraints

Helices are only defined if they have are long enough to make a complete turn of helix. This is 3, 4 and 5 residues for 3_10_, α and π helices respectively. For mixed helices the total length of the helix must be at least 4 residues. β-strands and poly-proline helices have a minimum length of 3 residues.

Ramachandran outliers will not be assigned to secondary structure classes by SEGNO due to the restrictions on φ and ψ. However, SEGNO checks for serious outliers and warns the user that secondary structure has not been assigned for this reason so that structures can be inspected if required. Outliers are defined according to the criteria of Lovell et al [27].

### Determination of secondary structure distortion

The program SSGEOM (SCL, unpublished) was used. This involved the generation of a secondary structural element in a standard reference frame, corresponding to each secondary structure type assigned by SEGNO. The lengths of β-strand, 3_10 _helix, α-helix and π-helix used were 2, 3, 4 and 5 residues, respectively. The standard secondary structure was superimposed onto the SEGNO-assigned secondary structure of the protein. The matrix required to superimpose the standard secondary structure was then applied to the known axis of the standard structure, and this axis was taken as the local axis of the secondary structure. The bend in a secondary structural element was defined as the angle between two of such local axes at a given residue.

### Determination of alignment accuracy

In order to determine the effect of differing assignments on secondary-structure guided sequence alignments, SEGNO assignments were compared with those from DSSP and structure-based alignments from the HOMSTRAD database. 285 2-member families were selected from HOMSTRAD. SEGNO and DSSP were used to assign secondary structures and environment-specific substitution tables [23,24] were derived using the SUBST program (Mizugichi, unpublished ). FUGUE [7] was used to derive a profile from one member of the family based on the secondary structure assignments, and to align this profile to the other member of the family. The resulting alignment was used as input to ProFit (Martin, A.C.R., , which uses the McLachlan algorithm [28]). Alignments were judged as being more accurate if they resulted in lower RMSD i.e. that the alignment derived from the profile match represents structural similarity.

### Model Set

For validation we have used a database of 500 structures of better than 1.8 Å resolution which has been developed for a study on the backbone torsion angles [27]. When HOMSTRAD families were used, 285 2 member families were chosen over range of sequence similarities (percentage identity between 15 and 45%).

## Authors' contributions

MVC and FC wrote the software and performed the analysis. SCL conceived and designed and coordinated the study and wrote the manuscript. All authors read and approved the manuscript.

## List of Abbreviations

RMSD – Root mean square deviation

## Figures and Tables

**Table 1:** Position of helix N-caps. The letter under the position number corresponds to the assignment of the residue relatively to the concerned helix. Thus the position 1 corresponds to the first helical residue and the N-cap residue should correspond to the position 0. Absolute counts and percentages are given.

Position of the N-cap	-1 C	0 C	1 H	2 H	3 H
Segno	8 (0.8%)	1049 (99.2%)	0 (0%)	0 (0%)	0 (0%)
Stride	29 (2,9%)	1016 (97.1%)	0 (0%)	0 (0%)	0 (0%)
DSSP	32 (3.1%)	1013 (96.9%)	0 (0%)	1 (0.1%)	1 (0.1%)

**Table 2:** C-caps of alpha helices: position of the first residue with φ > 0. The letter under the position number corresponds to the assignment of the residue relatively to the helix concerned. Thus the position -1 is the last helical residue and should correspond to the C-cap, while the position 0 must correspond to the first residue with a positive φ.

Position of the φ > 0 residue	-3 H	-2 H	-1 H	0 C	1 C	2 C	3 C
Segno	0 (0%)	0 (0%)	0 (0%)	2458 (81%)	277 (9,1%)	182 (6%)	119 (3,9%)
Stride	3 (0,1%)	0 (0%)	1 (0%)	2335 (84,8%)	183 (6,6%)	124 (4,5%)	107 (3,9%)
DSSP	13 (0.4%)	14 (0.5%)	30 (1%)	1911 (63,9%)	709 (23.7%)	193 (6,5%)	119 (4%)
